# GPNMB, LRRK2, and lysosome exocytosis in Parkinson’s

**DOI:** 10.1126/sciadv.aed8002

**Published:** 2025-12-17

**Authors:** Suzanne R. Pfeffer

**Affiliations:** Department of Biochemistry, Stanford University School of Medicine, Stanford, CA 94305-5307, USA and Aligning Science Across Parkinson’s, Chevy Chase, MD 20815 USA.

## Abstract

When two genes linked to increased Parkinson’s risk converge on a lysosome, LRRK2 mutation enhances lysosomal release of soluble GPNMB potentially contributing to synuclein pathology.

GPNMB (glycoprotein nonmetastatic melanoma protein B) is a type I transmembrane glycoprotein expressed in many cell types and tissues, including macrophages, microglia, osteoclasts, melanocytes, and some cancer cells. Prior work established that a portion of GPNMB is released from cellular membranes by ADAM family metalloproteases. In brain regions that are important in Parkinson’s disease (PD) (the substantia nigra and striatum), *GPNMB* is almost exclusively expressed in macrophage-like microglia and, to a lesser extent, in lysosome-rich astrocytes.

Genome-wide association studies have uncovered over 100 genome sequence variants that contribute to risk for Parkinson’s disease (PD), the second most common neurodegenerative disorder that affects approximately 6 million people worldwide. One risk allele near the *GPNMB* gene is linked to higher GPNMB expression in brain (*1*), and elevated GPNMB is also detected in PD patient plasma and cerebrospinal fluid (CSF) (*2*–*4*). Elevated GPNMB is not specific to PD: It is elevated in Alzheimer’s, amyotrophic lateral sclerosis, Gaucher’s and Niemann Pick C diseases, as well as in obesity and numerous cancers. Thus, understanding the role of GPNMB in health and disease is of great interest. In this issue of *Science Advances*, Bogacki *et al.* (*5*) characterize GPNMB in macrophages and highlight its role as a lysosomal protein that is delivered to the cell surface under conditions of lysosome stress; their work connects GPNMB with another important, PD-linked gene product, leucine-rich repeat kinase 2 (LRRK2) (*6*).

GPNMB is related to the melanocyte protein, PMEL; like PMEL, GPNMB is critical for melanosome formation and pigmentation in melanocytes. In humans, loss of GPNMB function is associated with skin pigmentation disorders such as amyloidosis cutis dyschromica that presents with mottled hyper- and hypopigmentation. Homology with PMEL and a role in melanosome function indicate that GPNMB is a protein of endo-lysosome–related organelles (such as melanosomes) that would be expected to undergo calcium-regulated exocytosis to mediate cell type–specific functions (*7*). Other examples of endo-lysosome–related organelles include secretory granules in cytotoxic T lymphocytes that release perforin and granzymes, and lamellar bodies in lung type II pneumocytes, that store and secrete surfactant to reduce alveolar surface tension.

Bogacki *et al.* (*5*) show that treatment of macrophages with the lysosomal damaging agent LLOME, the lysosomal V-ATPase inhibitor, bafilomycin A1, or the ionophore, nigericin, up-regulates GPNMB and triggers its secretion (Fig. 1). These treatments trigger endo-lysosome exocytosis (*8*), and the findings are consistent with GPNMB’s localization to the endocytic pathway, its activation by lysosome master transcription factors MITF and TFEB, and GPNMB’s reliance upon a dileucine signal in its cytoplasmic domain for such localization in multiple cell types. GPNMB showed increased colocalization with a bona fide lysosomal marker, LAMP1, after induction of lysosome stress, suggesting that in macrophages, it occupies a peri-nuclear, multivesicular endosome that fuses with lysosomes upon lysosome damage or stress.

**Fig. 1.**
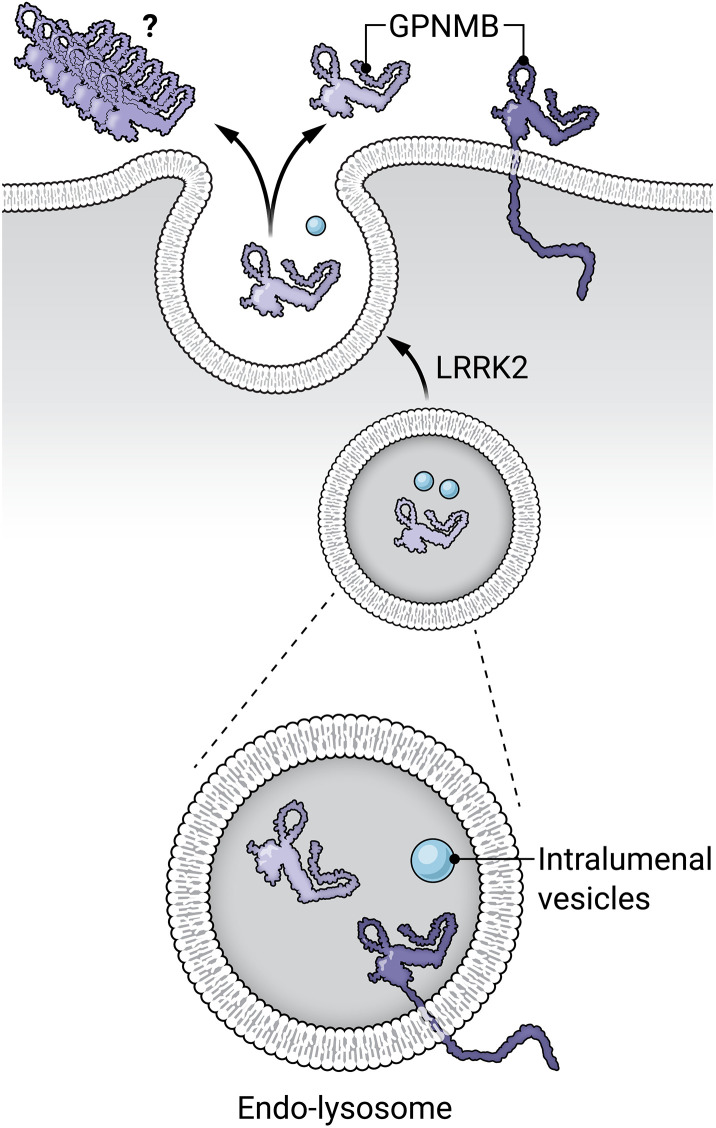
GPNMB is a membrane spanning glycoprotein that is delivered to endo-lysosomes. Upon endo-lysosome stress, soluble GPNMB will be released from endo-lysosomes into the extracellular space by a process catalyzed by Parkinson’s linked, activated LRRK2 kinase. GPNMB also binds α-synuclein and may influence α-synuclein pathology. The GPNMB model was created using AlphaFold 3. Credit: Austin Fisher, *Science Advances*.

After analyzing the Parkinson’s Progression Markers Initiative dataset, Bogacki *et al.* noted that *LRRK2* mutation carriers showed highly elevated GPNMB levels in their CSF, higher than controls and still higher than people with idiopathic PD or PD linked to *GBA* mutation. The *LRRK2* mutant CSF samples did not show a higher level of total lysosomal proteins, but this is likely because most lysosomal enzymes are soluble proteins that bear mannose 6-phosphate–terminating oligosaccharides that enable them to be recaptured by cells via cell surface mannose 6-phosphate–specific receptors. GPNMB would not carry such a recapture signal and thus would not be depleted from the CSF. [Shorter term assays of lysosomal enzyme release in cell culture (*8*) might not provide adequate time for such secreted lysosomal enzyme reuptake.] 

Why would *LRRK2* mutation carriers secrete extra GPNMB? LRRK2 plays an important but poorly understood role in the exocytosis of endo-lysosome–related organelles (*7*). Evidence for such a role comes from studies of urinary levels of the lysosome-specific lipid, bis(monoacylglycerol) phosphate (BMP), that are higher in *LRRK2* mutation carriers and decrease in mice, nonhuman primates, and patients treated with LRRK2 kinase inhibitors. In those studies, LRRK2 inhibitors are thought to block lysosome exocytosis in the kidney. LRRK2 inhibition also blocks exocytosis of endo-lysosome–related organelles in type II pneumocytes of the lung that secrete pulmonary surfactant; prolonged inhibition of this process causes pulmonary fibrosis, a side effect that is carefully monitored in ongoing LRRK2 inhibitor clinical trials. Especially in lysosome-rich macrophages that express high levels of LRRK2 (but also in other cell types), LRRK2 is recruited to damaged endo-lysosomes (*8*, *9*) and in some way contributes to endo-lysosome and endo-lysosome–related organelle exocytosis (*5*, *8*–*10*). Thus, hyperactive LRRK2 would increase exocytosis of soluble, endo-lysosome localized GPNMB.

LRRK2 kinase phosphorylates membrane-anchored Rab GTPases that are important for membrane trafficking, including endo-lysosome biogenesis and exocytosis (*6*). Rab3A, Rab8A, Rab10, and/or Rab27 have been reported to contribute to endo-lysosome exocytosis, and all (except Rab27) are LRRK2 substrates. But generic lysosomes do not generally have LRRK2 or Rabs on their surfaces, although Rab27, Rab32, and Rab38 are present on specialized endo-lysosome–related organelles (*7*). Rab phosphorylation has an unusual feature—it traps phosphorylated Rabs on the membrane where they are localized (or mislocalized) because they cannot be extracted by the “GDI” Rab recycling factor (*6*). Thus, any time LRRK2 is recruited to a membrane, phosphorylated substrate Rabs will accumulate there, unless they can encounter the Rab-specific PPM1H or PPM1M phosphatases.

Bentley-DeSousa *et al.* (*9*) have shown that lipidated GABARAP generated on the surface of damaged lysosomes recruits LRRK2 to lysosome surfaces. Once there, any mislocalized Rab3A, Rab8A, or Rab10 will become phosphorylated and trapped—and these phosphoRabs will accumulate, making it appear that LRRK2 is “activated.” Indeed, phosphoRabs also recruit LRRK2 to membranes as part of a feed-forward process. PhosphoRab8A and phosphoRab10 may then bind selectively to phosphoRab-specific, organelle motility–related JIP3, JIP4, and MyoVa proteins (*6*). Whether and how these (or other) phosphoRab-specific binding partners facilitate lysosome exocytosis is not yet known.

How would GPNMB elevation increase PD risk? As mentioned earlier, GPNMB is structurally related to PMEL, a protein needed for melanosome biogenesis. PMEL is processed by pro-protein convertase enzymes within the secretory pathway and delivered to a special class of multivesicular endosome/premelanosome (*7*). There, it is further processed and assembles into a fibrillar, amyloid matrix for the subsequent deposition of melanin. Note that GPNMB shares with PMEL a “polycystic kidney disease (PKD)” domain that, in PMEL, assembles into amyloid sheets onto which the melanin is deposited. Moreover, two of four upstream residues that are essential in PMEL for PKD aggregation are conserved in GPNMB. Membrane anchored, full-length GPNMB might be blocked from amyloid assembly, but protease-cleaved, soluble GPNMB containing the conserved PKD domain may form aggregates—indeed, insoluble GPNMB has been detected (*11*), although it is less likely to aggregate than PMEL due to GPNMB-specific glycosylation (*11*). GPNMB has also been reported to bind to α-synuclein (*2*). Although GPNMB appears to have been ruled out as an essential receptor for synuclein aggregate uptake in vivo (*3*), its possible tendency for aggregation and potential ability to interact with α-synuclein could facilitate Lewy body formation and other synuclein-driven pathological processes, especially in combination with pathogenic, hyperactive LRRK2.

PMEL is thought to localize to the intralumenal vesicles of multivesicular endosomes, together with the lysosome-specific BMP lipid. If GPNMB shares this localization, its up-regulation may interfere with BMP activation of important lysosomal enzymes such as PD-linked GBA1, the CLN5 BMP synthase, and the PLAG15 BMP hydrolase. Future experiments will be needed to clarify further the molecular composition of secreted GPNMB and its precise intra-endosomal localization and impact on BMP-activated enzymes. Finally, soluble, extracellular GPNMB has also been reported to bind to receptors including CD44, syndecan-4, integrins, and Na^+^/K^+^-ATPase, potentially influencing cell migration, immune modulation, and trophic support. Which of these (or other) binding interactions are relevant to GPNMB-associated neurodegeneration represent important areas for further study. Nevertheless, serum and CSF GPNMB levels may provide an important biomarker for target engagement in LRRK2 inhibitor clinical studies.

## References

[1] M. N. Murthy, C. Blauwendraat, S. Guelfi, J. Hardy, P. A. Lewis, D. Trabzuni, Increased brain expression of GPNMB is associated with genome wide significant risk for Parkinson’s disease on chromosome 7p15.3. Neurogenetics 18, 121–133 (2017). 28391543 10.1007/s10048-017-0514-8 PMC5522530

[2] M. E. Diaz-Ortiz, Y. Seo, M. Posavi, M. C. Cordon, E. Clark, N. Jain, R. Charan, M. D. Gallagher, T. L. Unger, N. Amari, R. T. Skrinak, R. Davila-Rivera, E. M. Brody, N. Han, R. Zack, V. M. Van Deerlin, T. F. Tropea, K. C. Luk, E. B. Lee, D. Weintraub, A. S. Chen-Plotkin, GPNMB confers risk for Parkinson’s disease through interaction with α-synuclein. Science 377, eabk0637 (2022). 35981040 10.1126/science.abk0637 PMC9870036

[3] R. Brendza, H. Lin, K. Stark, O. Foreman, J. Tao, A. Pierce, H. Ngu, K. Shen, A. E. Easton, T. Bhangale, D. Chang, B. Bingol, B. A. Friedman, Genetic ablation of Gpnmb does not alter synuclein-related pathology. Neurobiol. Dis. 159, 105494 (2021). 34464706 10.1016/j.nbd.2021.105494

[4] X. Qi, Z. She, X. Shi et al., Increased plasma GPNMB levels in patients with parkinson’s disease and cognitive impairment. Sci. Rep. 15, 20684 (2025). 40594632 10.1038/s41598-025-07415-6 PMC12214917

[5] E. C. Bogacki, G. Longmore, P. A. Lewis, S. Herbst, GPNMB is a biomarker for lysosomal dysfunction and is secreted via LRRK2-modulated lysosomal exocytosis. Sci. Adv. 11, eadv1434 (2025). 10.1126/sciadv.adv1434 PMC13142706 41406229

[6] S. R. Pfeffer, D. R. Alessi, “*Leucine-rich repeat kinase 2: Pathways to Parkinson’s disease. In Parkinson’s Disease*” in *Cold Spring Harbor Perspectives in Medicine* (Cold Spring Harbor Laboratory Press, ed. 2, 2025), pp. 133–150.10.1101/cshperspect.a04162040588344

[7] C. Delevoye, M. S. Marks, G. Raposo, Lysosome-related organelles as functional adaptations of the endolysosomal system. Curr. Opin. Cell Biol. 59, 147–158 (2019). 31234051 10.1016/j.ceb.2019.05.003 PMC6726539

[8] T. Eguchi, T. Kuwahara, M. Sakurai, T. Komori, T. Fujimoto, G. Ito, S.-I. Yoshimura, A. Harada, M. Fukuda, M. Koike, T. Iwatsubo, LRRK2 and its substrate Rab GTPases are sequentially targeted onto stressed lysosomes and maintain their homeostasis. Proc. Natl. Acad. Sci. U.S.A. 115, E9115–E9124 (2018). 30209220 10.1073/pnas.1812196115 PMC6166828

[9] A. Bentley-DeSousa, D. Clegg, S. M. Ferguson, LRRK2, lysosome damage, and Parkinson’s disease. Curr. Opin. Cell Biol. 93, 102482 (2025). 39983584 10.1016/j.ceb.2025.102482

[10] S. D. Palumbos, J. Popolow, J. Goldsmith, E. L. F. Holzbaur, Autophagic stress activates distinct compensatory secretory pathways in neurons. Proc. Natl. Acad. Sci. 122, e2421886122 (2025). 40623183 10.1073/pnas.2421886122 PMC12280970

[11] A. C. Theos, B. Watt, D. C. Harper, K. J. Janczura, S. C. Theos, K. E. Herman, M. S. Marks, The PKD domain distinguishes the trafficking and amyloidogenic properties of the pigment cell protein PMEL and its homologue GPNMB. Pigment Cell Melanoma Res. 26, 470–486 (2013). 23452376 10.1111/pcmr.12084 PMC3695043

